# Baseline Neutrophil-to-Lymphocyte Ratio Stratifies Early Trichoscopic Response to Platelet-Rich Plasma–Based Regimens in Non-Scarring Alopecia: A Real-World Cohort with Internal Validation Using an Interpretable Neural Network

**DOI:** 10.3390/life16040606

**Published:** 2026-04-06

**Authors:** Adelina Vrapcea, Sarmis-Marian Săndulescu, Eleonora Daniela Ciupeanu-Calugaru, Emil-Tiberius Traşcă, Dumitru Rădulescu, Patricia-Mihaela Rădulescu, Cristina Violeta Tutunaru, Sandra-Alice Buteica, Elena-Camelia Stănciulescu, Cătălina Gabriela Pisoschi

**Affiliations:** 1 Doctoral School, University of Medicine and Pharmacy of Craiova, 200585 Craiova, Romania; adelinavrapcea@yahoo.com; 2 Department of Surgery, University of Medicine and Pharmacy of Craiova, 200349 Craiova, Romania; sarmis.sandulescu@umfcv.ro (S.-M.S.); dr_radulescu_dumitru@yahoo.com (D.R.); 3 Department of Biology and Environmental Engineering, University of Craiova, 200585 Craiova, Romania; 4 Department of Pneumology, University of Medicine and Pharmacy of Craiova, 200349 Craiova, Romania; paty_miha@yahoo.com; 5 Department of Dermatology, University of Medicine and Pharmacy of Craiova, 200349 Craiova, Romania; cristina.tutunaru@umfcv.ro; 6 Pharmaceutical Chemistry Department, University of Medicine and Pharmacy of Craiova, 200349 Craiova, Romania; alice.buteica@umfcv.ro; 7 Department of Biochemistry, University of Medicine and Pharmacy of Craiova, St. Petru Rares, No. 2–4, 200433 Craiova, Romania; camelia.stanciulescu@umfcv.ro (E.-C.S.); catalina.pisoschi@umfcv.ro (C.G.P.)

**Keywords:** platelet-rich plasma, non-scarring alopecia, trichoscopy, neutrophil-to-lymphocyte ratio, systemic inflammation, prediction model, internal validation, explainable machine learning

## Abstract

Background/Objectives: Platelet-rich plasma (PRP)–based regimens are widely used in non-scarring alopecia, yet objective response is variable and clinic-ready predictors are lacking. We evaluated short-term trichoscopic outcomes in routine practice and tested whether baseline complete blood count–derived inflammatory status, quantified by the neutrophil-to-lymphocyte ratio (NLR), can stratify response under PRP-based therapy. Methods: We performed an ambispective observational cohort study (October 2024–October 2025) in an outpatient dermatology practice. The final analytic cohort included 129 patients allocated to four treatment groups: PRP alone (n = 54), PRP combined with microneedling-assisted Purasomes Hair & Scalp Complex (HCS50+, Dermoaroma; exosome-containing) (n = 33), PRP combined with microneedling-assisted Mesoaroma Hair Cocktail (scalp formulation; nutrient complex) (n = 24), and a nutrient complex alone (n = 18). Trichoscopy (FotoFinder ATBM; FotoFinder Systems GmbH, Bad Birnbach, Germany) was obtained at baseline (T1) and first follow-up (T2). Density response was defined as a ≥10% increase in total hair density and hair-cycle response as an anagen fraction increase ≥5 percentage points. Predictive analyses were prespecified and restricted to PRP-containing regimens, using logistic regression and a multilayer perceptron with repeated cross-validation for internal validation. Results: Across the full cohort (n = 129), total hair density and hair-cycle parameters improved from T1 to T2. In the PRP-containing subgroup (n = 111), baseline NLR strongly discriminated density responders (AUC 0.85, bootstrap 95% CI 0.77–0.91). In multivariable models, NLR remained independently associated with density response (OR 0.31 per 1-unit increase, 95% CI 0.20–0.48). Conclusions: In this cohort, baseline NLR was associated with discrimination of early trichoscopic response in PRP-based treatment of non-scarring alopecia. Using the Youden-derived cut-off (NLR = 2.202), patients with NLR > 2.202 had a higher risk of density non-response (72.1% vs. 4.7%), corresponding to a 15.49-fold increased failure risk in this cohort. These findings are exploratory and hypothesis-generating, and external validation and calibration are required before any routine clinical or decision-support use.

## 1. Introduction

Non-scarring alopecia represents one of the most frequent reasons for dermatology consultation and encompasses heterogeneous entities including androgenetic alopecia (AGA), telogen effluvium (TE), and alopecia areata (AA) [1,2,3,4]. Among these, AGA is the most prevalent and is associated with progressive follicular miniaturization, variable clinical course, and substantial psychosocial burden [1,2]. In routine practice, overlap with other non-scarring alopecia phenotypes may complicate patient counseling, treatment selection, and response assessment [3,4,5,6,7].

Current evidence-based management of AGA still relies on a limited number of approved options (topical minoxidil and oral finasteride), while many commonly used adjuvant or procedural therapies are supported by variable-quality evidence and are frequently adopted in “real-world” algorithms rather than standardized protocols [1,2,8]. This is particularly relevant for regenerative and device-assisted approaches such as platelet-rich plasma (PRP), microneedling, and exosome-based products, which are increasingly incorporated into individualized regimens despite limited protocol standardization and few head-to-head comparisons [2,8,9,10].

PRP has gained attention as an autologous, growth factor–enriched biologic that may enhance perifollicular microenvironmental signaling, support anagen maintenance, and improve hair parameters in AGA and other non-scarring alopecias [9,10,11]. Clinical studies and meta-analyses suggest that PRP can improve hair density and related trichoscopic endpoints, but reported effect sizes remain heterogeneous because of variation in preparation methods, treatment schedules, injection techniques, and outcome definitions [9,10,11,12,13]. This variability is clinically important because PRP is resource-intensive, typically requires repeated sessions, and yields inconsistent outcomes across patients in real-world settings [2,12,13].

Microneedling and exosome-containing products are also increasingly used as adjuncts in scalp regenerative practice, although supporting evidence, product characterization, and treatment protocols remain heterogeneous [14,15,16]. This evolving procedural landscape reinforces the need for pragmatic evidence reflecting real-world decision-making, where regimen choice is often guided by clinician judgment and patient preference rather than randomization.

A critical challenge across these interventions is outcome assessment. Trichoscopy and digital phototrichogram approaches provide non-invasive, repeatable, and quantitatively informative endpoints for objective monitoring over time [5,17]. Nevertheless, even with standardized imaging, clinicians still lack simple baseline tools to estimate the likelihood of meaningful improvement for a given patient—particularly in mixed real-world cohorts receiving multimodal treatment.

One attractive avenue for response stratification is the use of complete blood count (CBC)–derived systemic inflammation indices. Ratios such as the neutrophil-to-lymphocyte ratio (NLR), platelet-to-lymphocyte ratio (PLR), and systemic immune-inflammation index (SII) are inexpensive, widely available, and have been associated with inflammatory activity across dermatologic diseases [18,19]. Although AGA is not classically categorized as autoimmune, perifollicular inflammation and immune–vascular interactions are increasingly recognized as potential modifiers of disease expression and treatment responsiveness [1,2]. From a practical standpoint, CBC-derived indices may represent feasible baseline biomarkers for response stratification in procedural hair-loss clinics, where blood sampling is often already available as part of routine assessment.

Against this background, the key gap is not merely whether PRP regimens can improve hair metrics in selected patients, but whether routinely available baseline markers can help anticipate benefit under real-world clinical conditions. Addressing this gap could help clinicians better tailor regenerative regimens, set expectations, and rationalize follow-up intensity [20,21].

Therefore, the present study aimed to (1) describe real-world outcomes of scalp regenerative treatment protocols assessed with objective hair measurements and (2) explore whether baseline CBC-derived inflammation indices are associated with short-term clinical response, supporting pragmatic, hypothesis-generating stratification of patients more likely to benefit under real-world conditions.

## 2. Materials and Methods

### 2.1. Study Design, Setting, and Study Period

This ambispective observational study analyzed routinely collected clinical and laboratory data from patients evaluated and treated by board-certified dermatologists within a medical dermatology setting that includes an aesthetic and procedural dermatology service. Data collection included a retrospective component (clinical records available at the time of ethics approval) and a prospective component conducted under the framework of the approved research protocol. The study was performed between October 2024 and October 2025.

Retrospective data were extracted from existing medical records for patients treated before 4 April 2025, whereas prospective data were collected thereafter, following approval by the Ethics Committee of the University of Medicine and Pharmacy of Craiova (Approval No. 153/04.04.2025). To reduce bias related to the retrospective component, inclusion was restricted to patients meeting prespecified eligibility criteria, with paired standardized trichoscopic assessments at fixed study time points and sufficient baseline CBC differential data for prespecified index computation. Data extraction was limited to routinely documented variables using a predefined coding framework, and only one record per patient was included in the analytic dataset. All patients underwent standardized trichoscopic assessment at two predefined time points: baseline (T1, prior to therapy initiation) and follow-up (T2, after completion of the planned treatment course).

Follow-up evaluations (clinical, trichoscopic, and laboratory) were performed approximately 4 months after completion of the treatment course, reflecting routine clinical practice. A standardized 6-month follow-up was not uniformly available due to real-world scheduling constraints and patient adherence; therefore, analyses focused on the first post-treatment assessment available across the cohort.

### 2.2. Treatment Regimens and Rationale for Combined Approaches

Based on routine clinical practice and documentation in medical records, patients were categorized into four prespecified regimen groups: PRP monotherapy; PRP combined with Purasomes Hair & Scalp Complex (HCS50+, Dermoaroma Italy S.R.L., Rome, Italy; exosome-containing formulation); PRP combined with Mesoaroma Hair Cocktail (scalp formulation; Dermoaroma Italy S.R.L., Rome, Italy); and nutrient complex alone. Product details were derived from manufacturer documentation as used in routine practice; independent physicochemical characterization of the exosome content (e.g., particle sizing, cargo profiling) was not available beyond the manufacturer’s materials. PRP is widely used in non-scarring alopecia, and adjunctive approaches such as exosome-based preparations and nutrient formulations have been described in the literature and/or adopted in contemporary clinical practice as supportive strategies. In this real-world cohort, adjunct products (Purasomes HCS50+ or Mesoaroma Hair Cocktail (scalp formulation)) were applied according to their respective combined-regimen protocols as documented and verified from medical records, consistent with intended clinical use. These combinations were evaluated in a pragmatic, exploratory comparative context to examine whether trichoscopic outcomes differed across commonly used clinical combinations, acknowledging that regimen selection reflected routine clinical decision-making and patient preference rather than randomization. The final analytic cohort comprised 129 patients distributed across these four groups: PRP monotherapy (n = 54), PRP + exosomes (n = 33), PRP + nutrient complex (n = 24), and nutrient complex alone (n = 18).

### 2.3. Eligibility Criteria and Participant Flow

Patients were eligible if they had a clinical diagnosis of non-scarring alopecia managed with one of the prespecified regimens, had paired trichoscopic evaluations at T1 and T2 following the standardized acquisition protocol, and had baseline CBC differential data at T1 sufficient to compute prespecified inflammatory indices. Patients were excluded if they did not undergo the planned injection procedure(s) (including refusal after baseline evaluation), had incomplete critical data preventing endpoint computation, had documented autoimmune disease, or had evidence of pre-existing systemic inflammation at baseline when documented. HIV infection and chronic viral hepatitis, including hepatitis C, were considered exclusion conditions when documented, as these conditions may influence systemic inflammatory indices and confound the interpretation of CBC-derived biomarkers.

**Figure 1:**
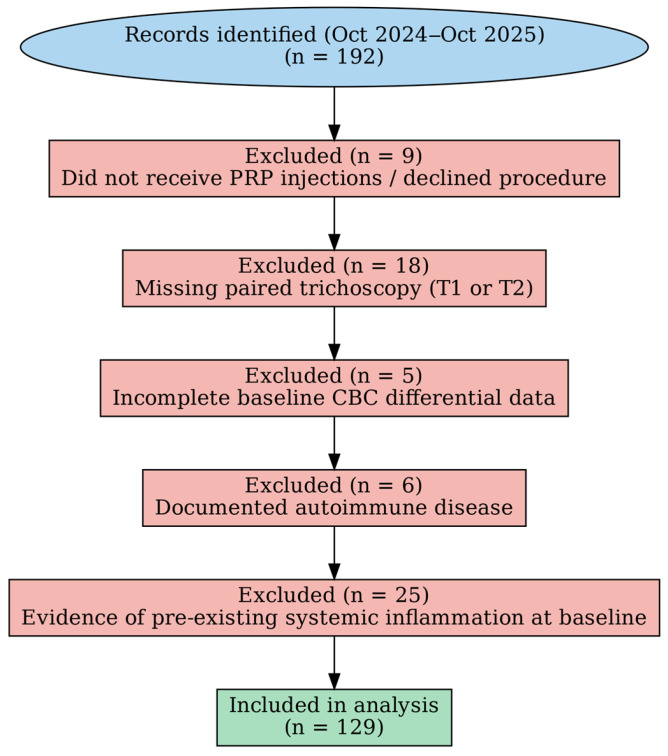
Patient selection flowchart.

During the study period, 192 records were screened. Exclusions comprised patients who did not receive PRP injections or declined the procedure (n = 9), lacked paired trichoscopy at baseline and follow-up (n = 18), had incomplete baseline CBC differential data required for index computation (n = 5), had documented autoimmune disease (n = 6), or presented evidence of pre-existing systemic inflammation at baseline (n = 25). The final analytic cohort included 129 patients (Figure 1).

Primary statistical analyses used a complete-case approach; within machine learning pipelines, any missingness in non-critical predictors was handled within cross-validation to prevent information leakage. These measures were intended to improve comparability across records and to limit bias arising from heterogeneous documentation in the retrospective component.

### 2.4. Data Sources, Variables, and Coding

Demographic and clinical covariates were extracted from routine clinical documentation and standardized patient questionnaires routinely used in the clinic, without altering routine clinical care. Variables included sex, BMI, smoking status, residence, education level, and concomitant dietary supplementation, when documented. Information on supplementation was collected as part of routine clinical assessment; however, supplementation was not standardized by study protocol and was not modeled as a primary exposure. Binary variables were encoded as 0 = no and 1 = yes. Sex was encoded as 0 = male and 1 = female. Residence was encoded as 0 = rural and 1 = urban.

Laboratory parameters were extracted from routine complete blood count reports. Leukocyte count parameters were available but were not included as standalone variables; analyses focused on prespecified inflammatory indices computed uniformly across the cohort.

When available, laboratory testing (CBC with differential) was repeated at follow-up (T2), approximately 4 months after treatment completion, using the same routine laboratory standards as at baseline.

### 2.5. CBC-Derived Inflammatory Indices

CBC-derived inflammatory indices were computed at baseline (T1) and, when available, at follow-up (T2), using platelet counts and leukocyte differential fractions. The neutrophil-to-lymphocyte ratio (NLR) was defined as neutrophils divided by lymphocytes, and the derived NLR (dNLR) as neutrophils divided by (100 − neutrophils). The monocyte-to-lymphocyte ratio (MLR) was defined as monocytes divided by lymphocytes, and the platelet-to-lymphocyte ratio (PLR) as platelets divided by lymphocytes. The systemic immune-inflammation index (SII) was computed as platelets multiplied by (neutrophils/lymphocytes). The systemic inflammation response index (SIRI) was computed as (neutrophils × monocytes)/lymphocytes, and the aggregate index of systemic inflammation (AISI) as (neutrophils × monocytes × platelets)/lymphocytes. Additionally, MCVL was computed as MCV/lymphocytes, and IIC as (MCV × RDW × neutrophils)/(lymphocytes × 1000). All indices were implemented according to a prespecified analysis codebook, using laboratory units recorded in the medical charts.

### 2.6. Trichoscopy Acquisition Protocol and Outcome Definitions

Trichoscopy was performed at T1 and T2 using a FotoFinder ATBM system (FotoFinder Systems GmbH, Bad Birnbach, Germany). Four standardized scalp regions were assessed, namely frontal, bitemporal, vertex, and parietal. Image acquisition followed a fixed protocol consisting of four images at baseline and four images at follow-up, with one image per region at each time point, aiming to replicate region and acquisition settings across visits.

The primary endpoint was the change in total hair density (hairs/cm^2^) from T1 to T2. Secondary endpoints included mean hair shaft thickness (mm), anagen and telogen fractions, terminal/vellus hair density, and other trichoscopic unit metrics where available. For predictive analyses, responder definitions were prespecified pragmatically as operational thresholds for early trichoscopic improvement: a density responder was defined by a ≥10% increase in hair density (T2 vs. T1), and a hair-cycle responder by a ≥5 percentage-point increase in anagen fraction (T2 vs. T1). These cut-offs were selected to provide clinically interpretable and objectively measurable early-response markers within the first routine follow-up window (approximately 4 months), rather than as universally validated response definitions derived from formal guidelines.

The follow-up trichoscopic assessment (T2) represented the first post-treatment evaluation available within routine clinical practice, typically performed at approximately 4 months.

Within this early follow-up context, a 10% increase in total hair density was considered a clinically meaningful directional improvement, while acknowledging that longer follow-up is required to determine durability and broader patient-level benefit.

### 2.7. PRP Administration and Adjunct Protocols

Platelet-rich plasma (PRP) was prepared from autologous peripheral blood using a closed PRP system (REGEN Lab SA, Le Mont-sur-Lausanne, Switzerland) and PRP Newlife ACS tubes (10 mL; 10 tubes/box; REGEN Lab SA, Le Mont-sur-Lausanne, Switzerland). Blood was processed by centrifugation for 7 min at 4000 rpm, according to the routine clinical protocol and the PRP system instructions, yielding a platelet-enriched plasma fraction suitable for intradermal administration. No exogenous activator (e.g., calcium chloride or thrombin) was added, and the PRP fraction was not gelified; the platelet-enriched plasma obtained after centrifugation was used as prepared for intradermal administration.

PRP was administered via intradermal scalp injections using 32-gauge needles, at an approximate depth of 2–4 mm. The total injected PRP volume was 5–6 mL, distributed across the frontal, bitemporal, vertex, and parietal scalp regions according to scalp surface area and clinical severity.

Adjunct products (an exosome-containing preparation or a nutrient complex) were used only in the corresponding combined-regimen groups and were applied using a microneedling pen device to facilitate transdermal delivery, following the combined-regimen protocol.

Microneedling-assisted application followed a standardized scalp protocol consistent with published approaches, using needle depths individualized to scalp thickness (typically 0.5–1.5 mm) and aiming for uniform erythema with minimal pinpoint bleeding, while avoiding excessive tissue trauma.

All protocols reflected real-world clinical use; regimen selection followed routine clinical decision-making and patient preference rather than randomization. All procedures were performed by board-certified dermatologists or under dermatologist supervision.

Post-procedure scalp-care recommendations and scalp-care products were provided as part of routine clinical practice; however, these measures were not standardized by the study protocol across all patients, and detailed product-level scalp-care data were not collected in a standardized analytic format.

### 2.8. Statistical Analysis

Continuous variables are presented as median (interquartile range, IQR) due to non-normal distributions. Within-group T1–T2 comparisons were performed using the Wilcoxon signed-rank test. Paired effect sizes were quantified using Cohen’s dz for paired differences and rank-based r (z/√n). Between-group comparisons of change scores were assessed using the Kruskal–Wallis test, followed by Holm-adjusted Mann–Whitney U tests when appropriate. Pairwise effect sizes were quantified using Cliff’s delta. For predictive inference, multivariable logistic regression models estimated odds ratios with 95% confidence intervals, adjusting for prespecified covariates (sex, BMI, smoking status, and treatment subtype). Discrimination was assessed using ROC AUC, and uncertainty was quantified using 1000× bootstrap resampling. A two-sided *p* < 0.05 was considered statistically significant. Statistical analyses and modeling were performed in Python (version 3.12.0) using NumPy (version 2.3.5), pandas (version 2.3.3), SciPy (version 1.16.3), statsmodels (version 0.14.6), scikit-learn (version 1.8.0), matplotlib (version 3.10.8), joblib (version 1.5.3), and graphviz (version 0.21).

### 2.9. Explainable Machine Learning: Modeling Strategy, Implementation, and Reproducibility

Prediction analyses were restricted to PRP-containing regimens to address the clinically relevant question of response stratification among patients receiving PRP-based therapy. Two classifiers were trained and compared, namely regularized logistic regression and a multilayer perceptron as a compact non-linear model. Because the predictive sample was modest in size, the multilayer perceptron was intentionally restricted to a compact architecture and a deliberately minimal feature set, and its role was exploratory rather than confirmatory. Model evaluation used repeated stratified 10-fold cross-validation repeated 10 times (10 × 10 repeated cross-validation), and performance was summarized as mean ± SD for ROC AUC, F1-score, and balanced accuracy. These design choices were intended to reduce overfitting risk; however, given the sample size, optimistic performance estimates and model instability cannot be fully excluded. Preprocessing was implemented using scikit-learn pipelines to prevent information leakage and included imputation when needed (median for numeric predictors and most frequent for categorical predictors), scaling of numeric features, and one-hot encoding of categorical features when applicable. Explainability included global feature relevance using permutation importance and local, case-level interpretability using LIME-style surrogate explanations based on locally perturbed samples.

## 3. Results

### 3.1. Cohort and Baseline Characteristics

A total of 129 patients were included in the final analysis and were allocated to four treatment regimens: PRP monotherapy (n = 54), PRP combined with exosomes (n = 33), PRP combined with a nutrient complex (n = 24), and nutrient complex alone (n = 18). Baseline demographic variables, baseline trichoscopic parameters, and complete blood count–derived inflammatory indices are summarized by treatment group in Table 1.

Across regimens, baseline trichoscopic severity was broadly comparable, including baseline total hair density, mean hair shaft thickness, and hair-cycle fractions (anagen and telogen).

**Figure 2:**
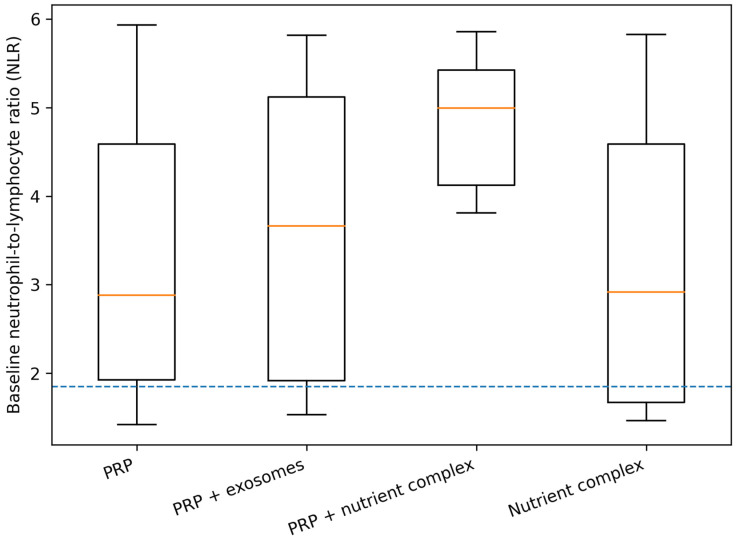
Baseline NLR distribution by treatment group.

Baseline systemic inflammatory status, as reflected by the neutrophil-to-lymphocyte ratio (NLR), showed variability between treatment regimens. The distribution of baseline NLR values across groups is visualized in Figure 2, which also indicates the pragmatic translational threshold (NLR = 1.85) used for subsequent stratified analyses.

Overall, these baseline data provide the descriptive clinical context for evaluating within-group changes from T1 to T2 and for assessing the prognostic value of NLR in PRP-containing regimens.

### 3.2. Within-Group Trichoscopic Changes

Within each regimen, paired trichoscopic parameters were compared between baseline (T1) and follow-up (T2). Across all groups, total hair density increased from T1 to T2, accompanied by a favorable shift in hair-cycle composition, with higher anagen fractions and lower telogen fractions at follow-up (Table 2).

Because multiple outcomes were evaluated across several subgroups, within-group T1–T2 analyses should be interpreted as exploratory; emphasis is placed on effect sizes, uncertainty, and consistency of direction rather than on isolated *p*-values.

**Figure 3:**
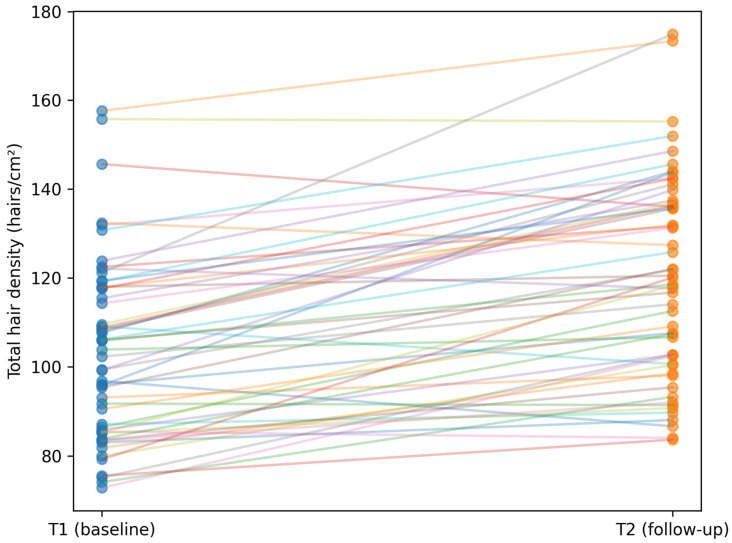
Paired change in total hair density from baseline (T1) to follow-up (T2) in the PRP monotherapy group. Each line represents one patient.

The magnitude of improvement varied between regimens, but the direction of change was consistent. In PRP monotherapy, the paired increase in hair density was pronounced and was accompanied by a clear anagen gain and telogen reduction. Individual paired trajectories for hair density in the PRP group are shown in Figure 3.

PRP combined with exosomes and PRP combined with a nutrient complex also demonstrated significant paired improvements in density and hair-cycle fractions. Nutrient complex monotherapy showed significant paired improvements in density and anagen fraction with a concordant telogen reduction. Across regimens, mean hair shaft thickness exhibited smaller changes and did not consistently reach statistical significance, suggesting that measurable short-term benefit was primarily driven by density and hair-cycle shifts rather than thickness-dominant effects.

**Figure 4:**
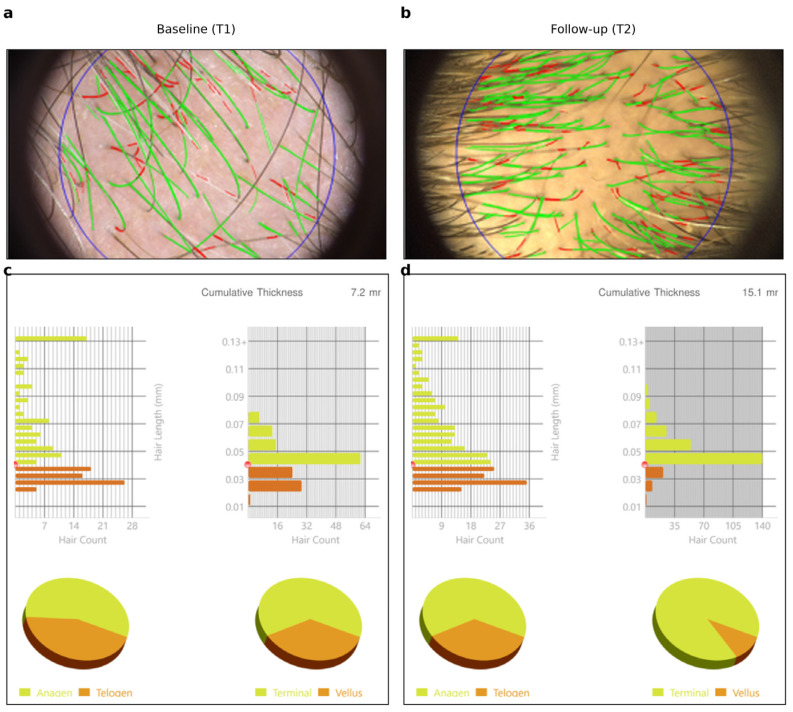
| Representative trichoscopic and quantitative imaging panels at baseline and follow-up in a PRP-containing regimen: (**a**,**b**) baseline (T1) and follow-up (T2) trichoscopic images from the same scalp area; (**c**,**d**) corresponding quantitative FotoFinder ATBM summary panels at T1 and T2.

To complement the quantitative analyses, we added a representative paired trichoscopic example from the prespecified PRP-containing subgroup. The case was selected from patients with paired baseline and follow-up documentation from the same scalp area acquired under standardized FotoFinder ATBM conditions, together with concordant improvement in the corresponding quantitative summary panel. This visual example is provided for illustration only and should be interpreted together with the predefined quantitative trichoscopic endpoints rather than as standalone evidence of efficacy. In this representative example, the follow-up panel shows visually increased shaft coverage and higher local hair density relative to baseline, while the accompanying FotoFinder summary panels illustrate the corresponding directional improvement in quantitative trichoscopic readouts (Figure 4).

### 3.3. Between-Group Comparisons of Change Scores

Between-group comparisons were performed on change scores (T2 − T1) to evaluate whether any regimen was associated with a larger magnitude of improvement in trichoscopic outcomes. Although median changes differed numerically across treatment groups, global rank-based testing did not identify statistically significant between-group differences for the primary endpoints. Specifically, the Kruskal–Wallis test was not significant for change in total hair density (*p* = 0.147), change in anagen fraction (*p* = 0.189), change in telogen fraction (*p* = 0.193), or change in mean hair shaft thickness (*p* = 0.329). Accordingly, post hoc pairwise comparisons did not yield statistically significant between-group differences after multiplicity control (Holm correction), and any true regimen-related contrasts—if present—are likely small relative to within-group variability in this retrospective dataset.

**Figure 5:**
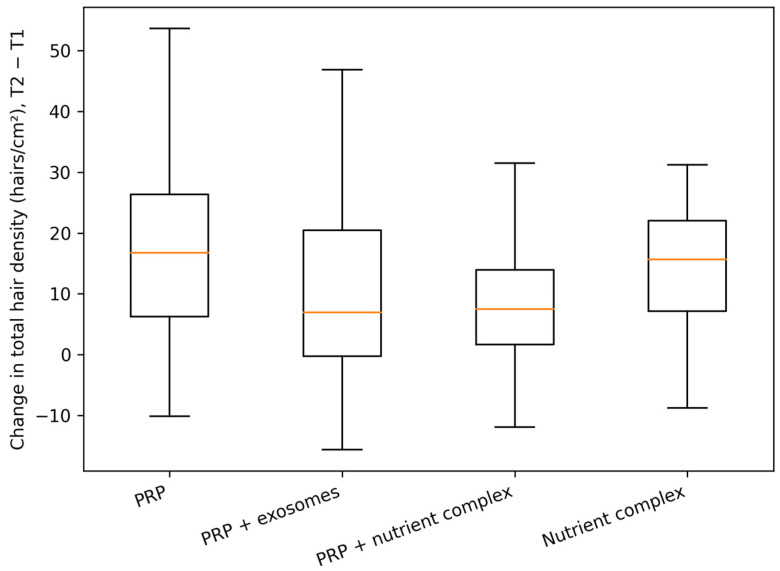
Change in total hair density (T2 − T1) stratified by treatment group. Boxes represent the interquartile range with the median line.

The distribution of density change across regimens is visualized in Figure 5 and demonstrates overlapping interquartile ranges between groups, consistent with the non-significant global test.

Taken together with the within-group paired analyses, these results support a consistent direction of improvement across regimens. However, the study was neither randomized nor powered for definitive head-to-head comparisons between treatment groups. Accordingly, any apparent between-regimen differences in trichoscopic change should be interpreted as exploratory only, because non-random treatment allocation, modest subgroup sizes, and confounding by indication may have influenced these contrasts.

### 3.4. NLR as a Predictor of Response to PRP (PRP-Containing Regimens)

Predictive analyses were prespecified for PRP-containing regimens (PRP, PRP + exosomes, PRP + nutrient complex; n = 111) to test whether baseline systemic inflammatory status, quantified by the neutrophil-to-lymphocyte ratio (NLR), could stratify clinically meaningful improvement after PRP-based intervention. Density response was defined as a ≥10% increase in total hair density at follow-up, yielding 60/111 (54.1%) responders.

**Figure 6:**
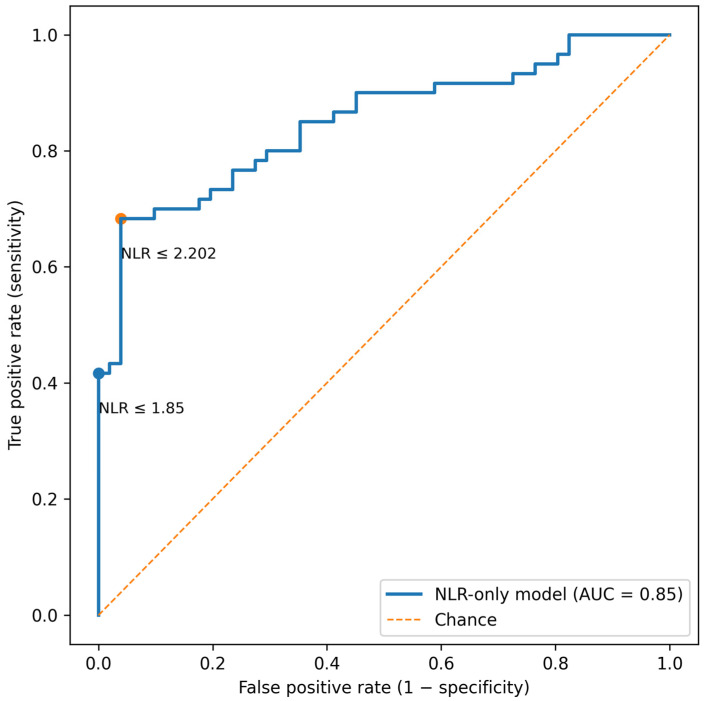
ROC curve for baseline NLR predicting density response (≥10% increase) in PRP-containing regimens; AUC with bootstrap 95% CI. Operating points corresponding to NLR ≤ 1.85 and NLR ≤ 2.202 are annotated.

Baseline NLR showed strong discrimination for density response. In an NLR-only logistic model, the ROC curve demonstrated AUC = 0.850 with a bootstrap 95% CI 0.773–0.914 (Figure 6). This discrimination was mirrored by a clear separation in baseline NLR distributions: density responders had substantially lower baseline NLR than non-responders (median 2.041 [IQR 1.681–4.108] vs. 5.076 [IQR 4.245–5.409], respectively; *p* < 0.001). Together, these results indicate that higher baseline inflammatory tone is associated with a lower probability of density improvement under PRP-based therapy.

To assess whether NLR remained informative beyond measured covariates, we fitted a prespecified multivariable logistic regression model; regimen subtype was included as an adjustment variable and not intended for causal comparison due to non-random treatment allocation. Baseline NLR remained an independent predictor of density response after adjustment for treatment subtype, sex, BMI, and smoking status, with an odds ratio for response of 0.31 per 1-unit increase in NLR (95% CI 0.20–0.48; *p* < 0.001) (Table 3).

Regimen subtype was included as an adjustment variable rather than a causal contrast, because treatment allocation was non-randomized and may reflect confounding by indication and unmeasured baseline severity.

In practical terms, higher baseline NLR was associated with lower odds of achieving the prespecified density response threshold, even after adjustment for measurable clinical covariates. Associations involving regimen subtype should be interpreted cautiously, because treatment allocation was non-randomized and may reflect confounding by indication and unmeasured severity-related factors.

The predictive signal was consistent when the endpoint was reframed as hair-cycle improvement. Hair-cycle response was defined as an anagen fraction increase ≥ 5 percentage points; 26/111 (23.4%) met this responder definition. Hair-cycle responders again had lower baseline NLR than non-responders (median 1.914 [IQR 1.640–2.113] vs. 4.515 [IQR 3.548–5.252]; *p* < 0.001). In adjusted analysis, baseline NLR remained independently predictive of hair-cycle response (OR 0.28, 95% CI 0.16–0.49; *p* < 0.001) (Table 4).

The concordant results across density and hair-cycle endpoints support a stable association between baseline inflammatory status and PRP responsiveness rather than an outcome-specific artifact.

Given that NLR is biologically and mathematically related to other complete blood count–derived inflammatory composites, we examined whether alternative indices provided comparable discrimination for response in PRP-containing regimens. Several indices demonstrated similar ROC performance for both density and hair-cycle endpoints, consistent with shared inflammatory information content across these measures (Table 5).

This convergence suggests the robustness of the baseline inflammation–response relationship and indicates that NLR performs within the top tier of readily available CBC-derived markers.

Because a continuous biomarker is most useful clinically when it can support transparent decision thresholds, we evaluated two operational cut-offs for density response. The Youden-optimized threshold (NLR = 2.202) provided a high-specificity operating point (sensitivity 0.683, specificity 0.961) with strong positive predictive value (PPV 0.953) (Table 6). Importantly, this threshold also conveyed an interpretable risk message: when NLR > 2.202, the risk of density non-response was 49/68 (72.1%), whereas for NLR ≤ 2.202 the non-response risk was 2/43 (4.7%). This corresponds to a 15.5-fold higher risk of density failure for patients with NLR > 2.202 (risk ratio 15.49, 95% CI 3.97–60.45). In parallel, a pragmatic “rule-in” threshold NLR ≤ 1.85 achieved specificity 1.00 and PPV 1.00, with no observed density non-responders among patients meeting this criterion, albeit with reduced sensitivity (Table 6).

Together, these results support a cautious interpretation: lower baseline NLR was associated with a higher probability of early density response in this cohort, whereas NLR above the balanced threshold was associated with an increased likelihood of non-response. Any treatment-selection use should remain restricted to prospective, externally validated decision tools.

### 3.5. Multi-Index Screening and Explainable Machine Learning Analyses

To complement classical inference with a model-comparison framework, machine learning models were trained using a deliberately minimal feature set (baseline NLR, baseline trichoscopy, BMI, sex, and treatment subtype) and evaluated using repeated 10 × 10 cross-validation. Discrimination for density response was high with logistic regression (AUC 0.92 ± 0.07) and remained high with the multilayer perceptron (AUC 0.89 ± 0.10), with comparable F1-scores and balanced accuracy, indicating that most predictive signal was captured by a parsimonious feature set and that non-linear modeling provided limited incremental gain for the density endpoint (Table 7).

For hair-cycle response, the multilayer perceptron showed a modest improvement in discrimination (AUC 0.83 ± 0.13 vs. 0.81 ± 0.13), suggesting that non-linear interactions among baseline severity, regimen subtype, and inflammatory status may become more relevant when the endpoint is defined by hair-cycle dynamics (Table 7).

All predictive performance estimates reflect internal validation only and may be optimistic; external validation, calibration assessment, and decision-analytic evaluation are required before any clinical decision-support use.

**Figure 7:**
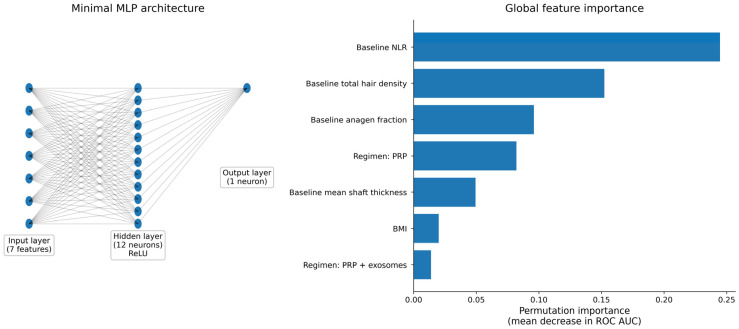
Explainable ML summary for density response prediction in PRP-containing regimens. Left panel: minimal MLP architecture. Right panel: global permutation importance.

Explainability analyses were then applied to assess whether model behavior remained clinically coherent and whether the learned decision rules aligned with the risk stratification implied by the NLR thresholds. Figure 7 summarizes the explainable machine-learning framework used for density response prediction in PRP-containing regimens, including the minimal multilayer perceptron (MLP) architecture and global permutation importance. In this context, permutation importance was expressed as the mean decrease in ROC AUC after random shuffling of each predictor, such that larger decreases indicated greater contribution to model discrimination.

NLR ranked among the most influential variables, consistent with the high failure-risk contrast observed when exceeding the Youden threshold (NLR > 2.202) and with the strong adjusted association in multivariable logistic regression. Baseline trichoscopic severity measures and treatment subtype also contributed to prediction, but NLR remained one of the most influential variables, supporting its potential role as a low-cost biomarker for pre-treatment stratification.

**Figure 8:**
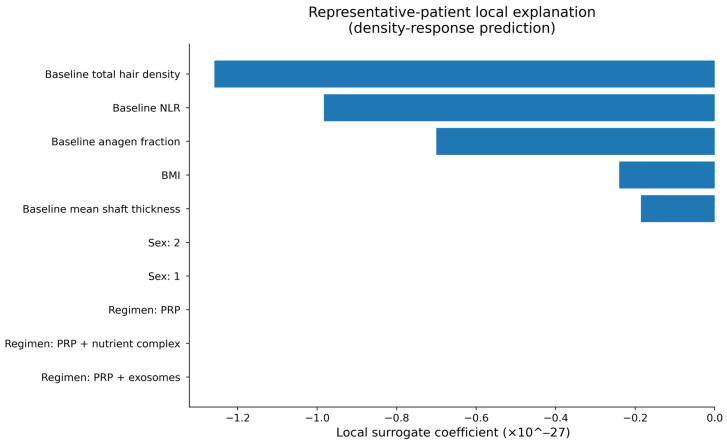
Local surrogate explanation for a representative PRP-containing patient, showing the variables contributing most to the predicted probability of density response.

To provide patient-level interpretability, local surrogate explanations were computed for representative individuals. As illustrated in Figure 8, a LIME-style local surrogate model was used to approximate the contribution of individual variables to the predicted probability of density response for a representative patient. The local explanation pattern consistently emphasized baseline NLR as a principal determinant of predicted response probability, alongside baseline hair density and regimen subtype. This suggests that higher baseline inflammatory status is associated with a lower predicted probability of response, while baseline trichoscopic severity may also influence the predicted likelihood of benefit.

**Figure 9:**
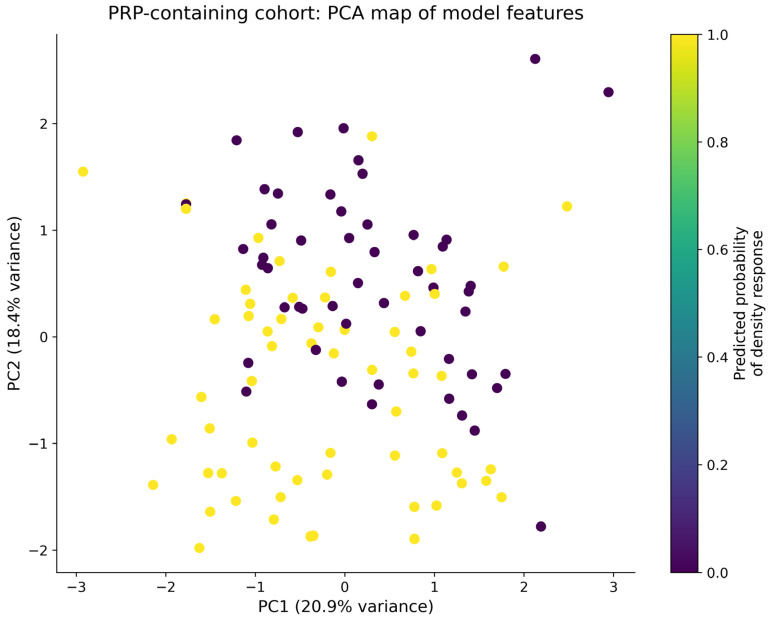
PCA-based patient map of the model feature space, colored by predicted probability of density response in PRP-containing regimens.

Finally, a PCA-based embedding of the model feature space was used to visualize patient positioning and predicted response probability. As shown in Figure 9, the preprocessed feature space was projected into two principal components and colored according to the model-predicted probability of density response. Patients formed a continuous gradient of predicted benefit rather than discrete clusters, consistent with a probabilistic stratification model.

Regions enriched for higher predicted response probability corresponded to lower NLR and more favorable baseline trichoscopic profiles. These findings suggest that baseline inflammatory status may help identify patients more likely to respond to PRP-based therapy.

## 4. Discussion

In this ambispective cohort from routine clinical practice, we evaluated whether a readily available systemic inflammatory marker, baseline neutrophil-to-lymphocyte ratio (NLR), can stratify early trichoscopic response to PRP-based scalp regimens, including PRP alone and PRP combined with microneedling-delivered adjunct preparations. Across regimens, objective trichoscopic parameters improved from baseline to follow-up, and baseline NLR was associated with discrimination of early density response in PRP-containing regimens. The association remained robust after adjustment for measurable clinical covariates and was supported by internally validated model comparisons and explainability analyses, suggesting that baseline inflammatory tone may contribute to inter-individual variability in measurable response to regenerative scalp procedures. The responder thresholds used in this study should be interpreted as pragmatic operational definitions of early objective improvement rather than universally established clinical-response standards. In particular, a 10% gain in hair density over approximately 4 months may be most appropriately viewed as an early trichoscopic signal of benefit, not as proof of durable long-term clinical success.

A potential practical implication is that NLR may help support exploratory response stratification and counseling in hair-loss clinics: patients with higher NLR may be less likely to show early trichoscopic gains under PRP-centered protocols, whereas lower NLR may indicate a more “permissive” regenerative context. Importantly, our results should be interpreted as internally validated evidence of association and stratification utility within the present cohort, rather than proof of generalizable predictive performance; considerations related to model development in limited datasets remain relevant [22], and independent external validation is required before any broader clinical application [23,24,25,26,27,28]. Using the Youden threshold (NLR = 2.202), baseline NLR > 2.202 identified a subgroup with a markedly increased risk of density non-response (15.49-fold higher failure risk in this cohort), which may be useful for expectation setting and for planning follow-up intensity in routine practice. Differences in baseline NLR between regimen groups likely reflect non-random treatment allocation (confounding by indication) rather than a regimen effect at baseline.

### 4.1. Inflammation and Hair Cycling: Possible Biological Basis of the NLR Association

Hair growth depends on a tightly regulated regenerative cycle (anagen–catagen–telogen), maintained by epithelial–mesenchymal signaling within the follicular unit and its niche, including dermal papilla function, perifollicular vasculature, and immune regulation [29,30]. Even when alopecia is clinically “non-inflammatory”, subtle shifts in the immune microenvironment can influence the transition between growth and regression phases and affect hair fiber production [30,31]. The concept of follicular immune privilege further supports biological plausibility: sustained anagen requires controlled immune quiescence around critical follicular compartments, and disruption of this balance can predispose to inflammatory amplification and impaired regenerative output [31].

Histopathologic literature in androgenetic alopecia (AGA) has long described perifollicular inflammatory infiltrates and “microinflammation”, linking low-grade inflammation to progressive remodeling and miniaturization [32,33]. While the inflammatory component of AGA is typically subtler than in alopecia areata, a persistent inflammatory background could still bias follicles toward less productive cycling and weaker structural recovery. In parallel, experimental evidence shows that pro-inflammatory cytokines can suppress hair growth and promote apoptosis of hair bulb keratinocytes—mechanisms consistent with reduced or delayed improvement on objective trichoscopic endpoints [34,35]. Regenerative pathways central to anagen induction and follicle morphogenesis, particularly Wnt/β-catenin signaling, are likewise sensitive to inflammatory and wound-healing contexts [36,37]. Taken together, these data provide a coherent biological frame for our observation: patients with higher baseline systemic inflammation (higher NLR) may be less likely to translate procedural regenerative signaling into early measurable hair gains.

### 4.2. PRP in Hair Regeneration: Why Baseline Inflammation Could Attenuate the Expected Benefit

PRP is widely used as an autologous, platelet-derived regenerative intervention intended to enhance angiogenesis, extracellular matrix remodeling, and survival/proliferation signaling within the follicular niche [38,39,40,41,42,43]. Clinical trials and comparative studies in AGA suggest that PRP can improve hair density and/or thickness, yet results are heterogeneous across protocols and cohorts [44,45,46,47]. A major reason is that PRP is not a uniform product: platelet dose, leukocyte content, preparation method, and activation strategies influence the balance between growth-promoting mediators and inflammatory/catabolic factors [40,41,42]. These compositional differences matter because follicular regeneration is highly context-dependent [29,30,31].

Our results are consistent with the idea that PRP response may depend partly on the host inflammatory background in which these cues are delivered. Elevated NLR may indicate a phenotype characterized by neutrophil predominance (greater oxidative/proteolytic inflammatory potential) and relative lymphopenia (less adaptive immune regulation), which could shift micro-injury responses toward prolonged inflammation rather than efficient resolution and constructive remodeling [37,48,49]. In this setting, PRP-driven anabolic signaling may be partially counteracted by inflammatory “noise” that disrupts growth factor gradients and matrix-mediated repair processes. This interpretation is consistent with broader tissue repair biology: regeneration requires an initial inflammatory phase, but excessive or unresolved inflammation can impair functional restoration [37].

### 4.3. Why NLR Is Clinically Useful Despite Being Non-Specific

NLR is not disease-specific, and leukocyte fractions can be influenced by transient infections, stress, smoking, metabolic inflammation, and medication use, and systemic inflammatory stress responses, which can shift leukocyte distributions and downstream immune function [50]. However, a non-specific marker may still be useful in prediction-oriented clinical workflows. NLR is a widely used index of systemic inflammation and stress that integrates two immune axes in a stable, routinely available ratio [49]. For procedural hair-loss practice, this is an advantage: NLR can be applied without specialized testing and can function as a response modifier rather than a diagnostic marker.

Evidence from alopecia areata supports the broader principle that CBC-derived inflammatory signatures can reflect clinically meaningful immune states relevant to follicular biology; several studies report associations between NLR (and related ratios) and disease severity or activity [51,52,53,54,55,56]. While AGA and AA differ mechanistically, these data reinforce the plausibility that systemic immune balance can influence follicle behavior and treatment response. In our context, the key point is pragmatic: baseline NLR may help explain why similar PRP-based regimens yield divergent early outcomes across patients, thereby supporting counseling, expectation setting, and potentially triage toward protocol intensification.

### 4.4. Adjunct Microneedling-Delivered Exosomes and Nutrient Complexes: Interpretation Through the Lens of Inflammation-Stratified Response

Adjunct “cell-free” approaches, including extracellular vesicles (EVs, often described as exosomes), aim to deliver concentrated bioactive signals that may complement PRP. Preclinical work indicates that PRP-derived exosomes and platelet-derived EVs can stimulate dermal papilla cell activity and promote hair follicle growth, with reported involvement of Wnt/β-catenin pathway signaling [57,58,59], which is central to follicular regeneration [36]. This provides a biologically plausible rationale for combined PRP + EV-based strategies, particularly when the regenerative response might otherwise be blunted by an unfavorable inflammatory background.

In routine practice, adjuncts are frequently delivered via microneedling to enhance transdermal delivery and provide diffuse exposure across the scalp. Microneedling itself can activate wound-healing cascades and has supportive evidence as an adjunct modality in AGA [14]. However, this creates an important interpretive nuance for our results: microneedling both facilitates delivery and induces controlled micro-injury; in patients with higher inflammatory tone (higher NLR), micro-injury could theoretically amplify innate inflammation, potentially diminishing net benefit unless the delivered adjunct effectively reinforces pro-regenerative signaling and resolution pathways [37,48]. Therefore, while our findings are compatible with an inflammation-stratified model of benefit, adjunct comparisons in observational cohorts should be interpreted cautiously due to confounding by indication, product heterogeneity, and the absence of randomization or power for definitive head-to-head inference.

### 4.5. Neural Network Modeling: What Our Results Support, and What They Do Not

Our internally validated neural network analyses were used to evaluate whether non-linear combinations of routine variables can support response discrimination in a real-world dataset. In principle, compact multilayer perceptrons can capture threshold effects and interactions (e.g., NLR behaving differently across baseline severity strata) that are difficult to specify a priori in purely linear models. This caution is particularly important in the present study because the predictive dataset was relatively small (n = 111), which increases the risk of overfitting for flexible models such as multilayer perceptrons despite repeated cross-validation and leakage-aware preprocessing. However, small-to-moderate clinical datasets are vulnerable to optimistic estimates if validation is not stringent; cross-validation can yield wide uncertainty, and model selection can introduce performance bias [24,25,26,27,28]. Accordingly, the correct interpretation of our modeling results is that neural models can extract clinically relevant signal from routine features under internal validation, supporting feasibility for stratification research, but they do not remove the need for external validation or calibration assessment prior to clinical implementation [23,24,25,26,27,28].

Overall, our findings support a cautious interpretation: internal validation indicates a reproducible stratification signal consistent with biological plausibility, which can inform future prospective designs (e.g., stratified enrollment or protocol selection). It does not support the claim that large samples are unnecessary; rather, it highlights how careful internal validation can generate actionable hypotheses that can then be tested in larger, independent cohorts [23,24,25,26,27,28].

Although calibration curves and decision-curve analysis would further strengthen assessment of model calibration and clinical utility, the present modeling framework was intended as internal, hypothesis-generating validation in a relatively small dataset. These evaluations are therefore more appropriately addressed in larger external-validation cohorts before any clinical implementation.

### 4.6. Limitations, Generalizability, and Next Steps

This study has several limitations that constrain causal inference and generalizability. The overall sample size was modest (n = 129), and the individual treatment groups were also relatively small (PRP, n = 54; PRP + exosomes, n = 33; PRP + nutrient complex, n = 24; nutrient complex alone, n = 18), which limits precision, reduces power for between-regimen comparisons, and increases uncertainty in subgroup-based inference. Because part of the cohort was assembled retrospectively from routine records, some degree of selection bias and residual information bias cannot be fully excluded despite the use of prespecified eligibility criteria, standardized trichoscopic endpoints, and restricted variable coding. First, regimen allocation was non-randomized, reflecting routine practice, and the study was not powered for definitive comparisons between treatment regimens; therefore, confounding by indication is possible and adjunct groups may differ systematically from PRP-alone recipients in ways that influence outcomes. As a result, any observed between-regimen differences in trichoscopic outcomes should be interpreted as exploratory rather than confirmatory. In addition, concomitant dietary supplementation was not standardized across patients and may have contributed to outcome heterogeneity. Post-procedure scalp-care practices and recommended scalp-care products were also not standardized across the cohort and were not captured in a fully standardized product-level format, which may have further contributed to outcome heterogeneity. Second, the cohort is single-center, and practice patterns, patient mix, and procedural technique may limit transportability. Third, follow-up at approximately 4 months captures early trichoscopic response but may not represent durability or later responders given the biology of hair cycling. Fourth, NLR is non-specific and potentially confounded by intercurrent conditions; repeated measurements and explicit adjustment for key contributors would strengthen inference. Fifth, PRP heterogeneity and incomplete compositional characterization remain important limitations; formal PRP classification and platelet dose reporting would improve comparability across studies. Finally, the predictive models were developed on a relatively small dataset and evaluated using internal validation only, which increases the risk of overfitting and optimistic performance estimates, particularly for the multilayer perceptron. In the absence of external validation in an independent cohort, the generalizability of the proposed predictive framework remains uncertain. Accordingly, these models should be considered hypothesis-generating rather than ready for routine clinical application, and external validation, recalibration, and calibration assessment are essential before any clinical use.

Because adjunct formulations were commercially sourced and characterized primarily through manufacturer documentation, the generalizability of adjunct-specific effects may be limited, and future studies should report standardized product specifications and independent characterization where feasible.

Future studies should therefore focus on multicenter cohorts with standardized PRP preparation/reporting frameworks, longer follow-up aligned to hair-cycle dynamics, repeated CBC sampling and confounder documentation, and external validation/recalibration of the predictive model using transparent prediction-model reporting standards. If confirmed, inflammation-stratified PRP pathways could support pragmatic personalization—using low-cost biomarkers to improve counseling and monitoring intensity—while maintaining appropriate caution about causality and generalizability. Mechanistically, these findings motivate prospective work linking systemic inflammatory tone to follicular cycling dynamics and regenerative signaling under PRP exposure, alongside standardized PRP characterization and platelet-dose reporting.

## 5. Conclusions

In a real-world cohort of non-scarring alopecia treated with PRP-based regimens, baseline neutrophil-to-lymphocyte ratio (NLR) was associated with early trichoscopic response discrimination. In PRP-containing regimens, an NLR threshold of 2.202 was associated with a 15.49-fold higher risk of density non-response when exceeded in this cohort. These findings are internally validated and hypothesis-generating only and should be interpreted as exploratory rather than practice-changing; external validation in independent multicenter cohorts, together with recalibration of predictive thresholds, is required before any routine clinical or decision-support implementation.

## Figures and Tables

**Table 1:** Baseline characteristics by treatment group.

Characteristic	PRP (n = 54)	PRP + Exosomes (n = 33)	PRP + Nutrient Complex (n = 24)	Nutrient Complex (n = 18)
Age (years)	37.00 [28.25, 44.00]	37.00 [30.00, 49.00]	39.50 [36.75, 46.00]	37.00 [32.25, 44.25]
BMI (kg/m^2^)	23.42 [20.86, 24.91]	24.16 [21.95, 24.69]	23.67 [21.84, 24.68]	22.91 [21.28, 24.73]
Baseline hair density (hairs/cm^2^)	103.06 [85.54, 117.79]	104.25 [94.02, 122.96]	105.44 [100.52, 118.99]	102.39 [96.05, 121.84]
Baseline mean thickness (mm)	0.04 [0.03, 0.04]	0.04 [0.03, 0.04]	0.03 [0.03, 0.04]	0.04 [0.03, 0.04]
Baseline anagen (%)	74.2 [71.8, 77.0]	74.6 [72.4, 78.2]	74.7 [71.4, 77.0]	76.0 [73.3, 81.0]
Baseline telogen (%)	25.8 [23.0, 28.2]	25.4 [21.8, 27.6]	25.4 [23.0, 28.6]	24.0 [19.0, 26.7]
Baseline NLR	2.88 [1.92, 4.59]	3.67 [1.92, 5.12]	5.00 [4.12, 5.42]	2.92 [1.67, 4.59]
Baseline dNLR	2.11 [1.47, 2.94]	2.61 [1.51, 3.10]	3.34 [2.69, 3.79]	2.08 [1.32, 3.09]
Baseline SII	772.54 [453.68, 1555.28]	1298.74 [435.15, 1787.10]	1540.08 [1222.61, 1744.06]	840.32 [444.66, 1443.50]
Platelets (10^3^/µL)	286.50 [244.50, 339.75]	318.00 [228.00, 358.00]	307.00 [277.25, 346.25]	280.50 [262.00, 308.00]
Female	21 (38.9%)	12 (36.4%)	7 (29.2%)	2 (11.1%)
Smoker	15 (27.8%)	19 (57.6%)	9 (37.5%)	5 (27.8%)
Alcohol use	10 (18.5%)	5 (15.2%)	7 (29.2%)	5 (27.8%)

Values are median [IQR] for continuous variables and n (%) for categorical variables.

**Table 2:** Paired trichoscopic outcomes (T1 vs. T2) by treatment group.

Treatment Group	Outcome	T1	T2	Δ (T2 − T1)	*p*	Cohen’s dz	Effect Size r
PRP (n = 54)	Hair density (hairs/cm^2^)	103.06 [85.54, 117.79]	119.31 [101.05, 136.72]	16.77 [6.21, 26.36]	<0.001	1.16	0.79
	Anagen fraction (%)	74.2 [71.8, 77.0]	78.2 [73.4, 82.8]	3.7 [1.1, 6.6]	<0.001	1.04	0.77
	Telogen fraction (%)	25.8 [23.0, 28.2]	21.9 [17.2, 26.6]	−3.7 [−6.6, −1.1]	<0.001	−1.04	−0.77
	Mean shaft thickness (mm)	0.04 [0.03, 0.04]	0.04 [0.03, 0.04]	0.00 [−0.00, 0.00]	0.040	0.23	0.28
PRP + exosomes (n = 33)	Hair density (hairs/cm^2^)	104.25 [94.02, 122.96]	119.48 [99.09, 135.71]	6.91 [−0.26, 20.42]	<0.001	0.73	0.63
	Anagen fraction (%)	74.6 [72.4, 78.2]	77.9 [74.3, 80.2]	2.4 [0.3, 3.7]	<0.001	1.07	0.76
	Telogen fraction (%)	25.4 [21.8, 27.6]	22.1 [19.8, 25.7]	−2.4 [−3.7, −0.3]	<0.001	−1.07	−0.76
	Mean shaft thickness (mm)	0.04 [0.03, 0.04]	0.04 [0.03, 0.04]	0.00 [−0.00, 0.01]	0.272	0.26	0.19
PRP + nutrient complex (n = 24)	Hair density (hairs/cm^2^)	105.44 [100.52, 118.99]	117.02 [104.93, 128.34]	7.51 [1.64, 13.96]	<0.001	0.80	0.77
	Anagen fraction (%)	74.7 [71.4, 77.0]	76.5 [71.6, 80.2]	2.1 [0.3, 3.2]	<0.001	0.77	0.73
	Telogen fraction (%)	25.4 [23.0, 28.6]	23.5 [19.8, 28.4]	−2.1 [−3.2, −0.3]	<0.001	−0.77	−0.73
	Mean shaft thickness (mm)	0.03 [0.03, 0.04]	0.03 [0.03, 0.04]	−0.00 [−0.00, 0.00]	0.509	−0.10	−0.13
Nutrient complex (n = 18)	Hair density (hairs/cm^2^)	102.39 [96.05, 121.84]	113.24 [105.70, 136.02]	15.63 [7.13, 22.01]	<0.001	1.20	0.85
	Anagen fraction (%)	76.0 [73.3, 81.0]	81.5 [77.8, 83.5]	2.5 [0.6, 5.8]	<0.001	0.98	0.91
	Telogen fraction (%)	24.0 [19.0, 26.7]	18.5 [16.5, 22.2]	−2.5 [−5.8, −0.6]	<0.001	−0.98	−0.91
	Mean shaft thickness (mm)	0.04 [0.03, 0.04]	0.04 [0.03, 0.04]	0.00 [−0.00, 0.00]	0.212	0.38	0.29

Values are median [IQR]. *p*-values are from Wilcoxon signed-rank tests.

**Table 3:** Multivariable logistic regression (PRP-containing): density responder (≥10% increase).

Predictor	OR	95% CI	*p*
Intercept	10.84	[0.15, 780.37]	0.275
PRP + exosomes vs. PRP	0.21	[0.06, 0.77]	0.019
PRP + nutrient complex vs. PRP	0.53	[0.14, 2.03]	0.354
Baseline NLR (per 1-unit increase)	0.31	[0.20, 0.48]	<0.001
Female sex	0.41	[0.12, 1.44]	0.163
BMI (per 1 kg/m^2^ increase)	1.14	[0.96, 1.37]	0.140
Smokers	1.26	[0.43, 3.67]	0.673

Odds ratios (OR) with 95% CI and *p*-values; reference regimen is PRP.

**Table 4:** Multivariable logistic regression (PRP-containing): hair-cycle responder (anagen increase ≥ 5 percentage points).

Predictor	OR	95% CI	*p*
Intercept	1.10	[0.01, 141.95]	0.968
PRP + exosomes vs. PRP	0.44	[0.11, 1.71]	0.236
PRP + nutrient complex vs. PRP	0.90	[0.15, 5.37]	0.910
Baseline NLR (per 1-unit increase)	0.28	[0.16, 0.49]	<0.001
Female sex	1.03	[0.25, 4.16]	0.967
BMI (per 1 kg/m^2^ increase)	1.12	[0.91, 1.38]	0.304
Smoker	1.18	[0.31, 4.51]	0.807

Odds ratios (OR) with 95% CI and *p*-values; reference regimen is PRP.

**Table 5:** ROC performance for selected inflammatory indices in PRP-containing regimens.

Outcome	Index	AUC	Direction	Cut-Off	Sensitivity	Specificity	n	n_pos
Density responder (≥10% increase)	NLR	0.85	low	2.202	0.683	0.961	111	60
Density responder (≥10% increase)	dNLR	0.83	low	1.817	0.683	0.961	111	60
Density responder (≥10% increase)	SII	0.85	low	1222.117	0.733	0.922	111	60
Density responder (≥10% increase)	PLR	0.84	low	1585.106	0.733	0.922	111	60
Density responder (≥10% increase)	MCVL	0.86	low	334.737	0.683	0.961	111	60
Density responder (≥10% increase)	IIC	0.87	low	2.800	0.667	0.980	111	60
Hair-cycle responder (anagen increase ≥ 5 pp)	NLR	0.85	low	2.202	0.923	0.776	111	26
Hair-cycle responder (anagen increase ≥ 5 pp)	dNLR	0.86	low	2.413	0.962	0.753	111	26
Hair-cycle responder (anagen increase ≥ 5 pp)	SII	0.85	low	621.115	0.923	0.776	111	26
Hair-cycle responder (anagen increase ≥ 5 pp)	PLR	0.86	low	970.492	0.923	0.776	111	26
Hair-cycle responder (anagen increase ≥ 5 pp)	MCVL	0.84	low	334.737	0.923	0.776	111	26
Hair-cycle responder (anagen increase ≥ 5 pp)	IIC	0.86	low	2.846	0.923	0.776	111	26

Direction indicates whether lower (low) or higher (high) index values predict response; cut-offs are Youden-optimized.

**Table 6:** Translational NLR thresholds for density response (PRP-containing).

NLR Threshold	Sensitivity	Specificity	PPV	NPV	TP/n	FP
1.85	0.383	1.000	1.000	0.580	23/111	0
2.202	0.683	0.961	0.953	0.721	41/111	2

Density response was defined as ≥10% increase in total hair density. “Test positive” indicates predicted responder (NLR ≤ threshold).

**Table 7:** Machine learning performance (repeated 10 × 10 cross-validation).

Model	Endpoint	AUC (Mean ± SD)	F1 (Mean ± SD)	Balanced Acc. (Mean ± SD)
Logistic regression (minimal)	Density responder	0.92 ± 0.07	0.82 ± 0.10	0.80 ± 0.11
Logistic regression (minimal)	Hair-cycle responder	0.81 ± 0.13	0.40 ± 0.27	0.63 ± 0.16
MLP (minimal, L-BFGS)	Density responder	0.89 ± 0.10	0.82 ± 0.10	0.81 ± 0.11
MLP (minimal, L-BFGS)	Hair-cycle responder	0.83 ± 0.13	0.49 ± 0.27	0.68 ± 0.16

Minimal feature set (baseline NLR, BMI, baseline trichoscopy, sex, and treatment subtype); values are mean ± SD.

## Data Availability

The data supporting the findings of this study are not publicly available due to ethical and privacy restrictions. Anonymized data are available from the corresponding author upon reasonable request, subject to institutional and ethical approval.

## Abbreviations

The following abbreviations are used in this manuscript:

AGA	androgenetic alopecia
AISI	aggregate index of systemic inflammation
AUC	area under the curve
BMI	body mass index
CBC	complete blood count
CI	confidence interval
dNLR	derived neutrophil-to-lymphocyte ratio
EV	extracellular vesicle
IIC	index of inflammation composite
IQR	interquartile range
LIME	local interpretable model-agnostic explanations
MCV	mean corpuscular volume
MCVL	mean corpuscular volume-to-lymphocyte ratio
MLP	multilayer perceptron
NLR	neutrophil-to-lymphocyte ratio
NPV	negative predictive value
OR	odds ratio
PCA	principal component analysis
PLR	platelet-to-lymphocyte ratio
PPV	positive predictive value
PRP	platelet-rich plasma
RDW	red cell distribution width
ROC	receiver operating characteristic
SII	systemic immune-inflammation index
SIRI	systemic inflammation response index
TE	telogen effluvium.
